# A microplanning model to improve door-to-door health service delivery: the case of Seasonal Malaria Chemoprevention in Sub-Saharan African villages

**DOI:** 10.1186/s12913-020-05972-2

**Published:** 2020-12-07

**Authors:** André Lin Ouédraogo, Julie Zhang, Halidou Tinto, Innocent Valéa, Edward A. Wenger

**Affiliations:** 1 Institute for Disease Modeling, Bill and Melinda Gates Foundation, 500 5th Ave N, Seattle, WA 98109 USA; 2 Department of Mathematics and Statistics, University of Washington, Seattle, WA USA; 3 Department of Statistics, Stanford University, Palo Alto, CA USA; 4 Institut de Recherche en Sciences de la Santé, Clinical Research Unit of Nanoro, Nanoro, Burkina Faso

**Keywords:** Malaria, Microplanning, Seasonal malaria chemoprevention, SMC, Door-to-door, Model, Community health worker, CHW, Satellite imagery, Burkina Faso

## Abstract

**Background:**

Malaria incidence has plateaued in Sub-Saharan Africa despite Seasonal Malaria Chemoprevention’s (SMC) introduction. Community health workers (CHW) use a door-to-door delivery strategy to treat children with SMC drugs, but for SMC to be as effective as in clinical trials, coverage must be high over successive seasons.

**Methods:**

We developed and used a microplanning model that utilizes population raster to estimate population size, generates optimal households visit itinerary, and quantifies SMC coverage based on CHWs’ time investment for treatment and walking. CHWs’ performance under current SMC deployment mode was assessed using CHWs’ tracking data and compared to microplanning in villages with varying demographics and geographies.

**Results:**

Estimates showed that microplanning significantly reduces CHWs’ walking distance by 25%, increases the number of visited households by 36% (*p* < 0.001) and increases SMC coverage by 21% from 37.3% under current SMC deployment mode up to 58.3% under microplanning (*p* < 0.001). Optimal visit itinerary alone increased SMC coverage up to 100% in small villages whereas in larger or hard-to-reach villages, filling the gap additionally needed an optimization of the CHW ratio.

**Conclusion:**

We estimate that for a pair of CHWs, the daily optimal number of visited children (assuming 8.5mn spent per child) and walking distance should not exceed 45 (95% CI 27–62) and 5 km (95% CI 3.2–6.2) respectively. Our work contributes to extend SMC coverage by 21–63% and may have broader applicability for other community health programs.

**Supplementary Information:**

The online version contains supplementary material available at 10.1186/s12913-020-05972-2.

## Background

Malaria remains the foremost health challenge in Sub-Saharan Africa [1]. Recent data showed that globally, progress in reducing malaria burden has stalled, especially in high-burden countries [1] urging the World Health Organization (WHO) to launch the country-led high burden to high impact (HBHI) approach. The goal of the HBHI is to bring the 11 highest burden countries, 10 in Sub-Saharan Africa plus India, back on track to achieve WHO Global Technical Strategy’s milestone which aims to reduce incidence by at least 75% by 2025 [2]; but that is unlikely to succeed unless key burden reduction strategies such as seasonal malaria chemoprevention (SMC) are revisited to maximize impacts.

Clinical trials reported that SMC prevent approximately 80% of malaria episodes among treated children [3–5]. At the global scale, modeling studies suggest that millions of malaria cases and thousands of deaths could be averted if SMC delivery on the ground was successful [6]. With regard to SMC deployment, studies comparing fixed-location versus door-to-door suggest the latter as most effective with respect to coverage [7–9]. After its recommendation by WHO in 2012, SMC was deployed in 2014, mostly in countries across the Sahel and Sahel sub-region where more than 60% of clinical malaria concentrate within 3-to-4 consecutive months. Nevertheless, recent data showed that SMC in its programmatic phase is failing as progress in reducing incidence has plateaued to date despite its introduction [1].

One likely reason SMC is not properly working under real-world conditions is to be associated to its poor delivery in the community. In Mali, the average of SMC coverage in 2016 as reported from seven surveys was 53% [10]. In Burkina Faso, post-campaign coverage estimates using SMC cards and parents’ statements showed that only a small fraction of children (32%) received all SMC doses over four consecutive rounds [11]. However, for SMC to reach the desired cases reduction, we must see high coverages above 90–95% over successive seasons [6, 12]. Multiple logistic constraints and shortcomings in SMC deployment including CHW ratio per capita [11], excess time loss during treatment [5], and importantly missed households or settlements as reported during Polio vaccination [13–15] likely contribute to lower SMC coverage.

A country’s microplanning strategy can be a complex and difficult process leaving activities more often unoptimized for impact.

Here we develop a microplanning model to predict CHW’s performance during door-to-door health service delivery and review opportunities to optimize and standardize SMC deployment that may contribute leverage its potential in preventing malaria episodes and accelerate malaria burden reduction toward 2025. The model utilizes population raster data on demographics (family sizes, household geolocations) to assess treatment duration and door-to-door travel times allowing for quantification of the CHW’s time investment that is convertible to actual SMC coverage and unmet needs. We then use the model to assess SMC coverage under its current deployment mode and to predict microplanning achievements in African remote villages.

## Methods

### Study site

Burkina Faso reported approximately 12 million of malaria cases and 4000 deaths in 2018 [1]. Malaria transmission is intense and seasonal [16, 17] and despite SMC, clinical malaria remains on the rise [1]. The health and demographic surveillance site (HDSS) of Nanoro in the centre west provides rich household survey data suitable for microplanning studies [18]. Three villages (Soaw, Rakolo, Mogdin) with different characteristics (geographic or demographics) were selected to test the potential of microplanning in optimizing SMC deployment. Population raster data were extracted to compare villages’ characteristics and results are presented in the results’ section.

Census data were obtained from the HDSS and incidence data from national’s District Health Information Software 2 platforms **(**DHIS2).

### Microplanning model

**Fig. 1:**
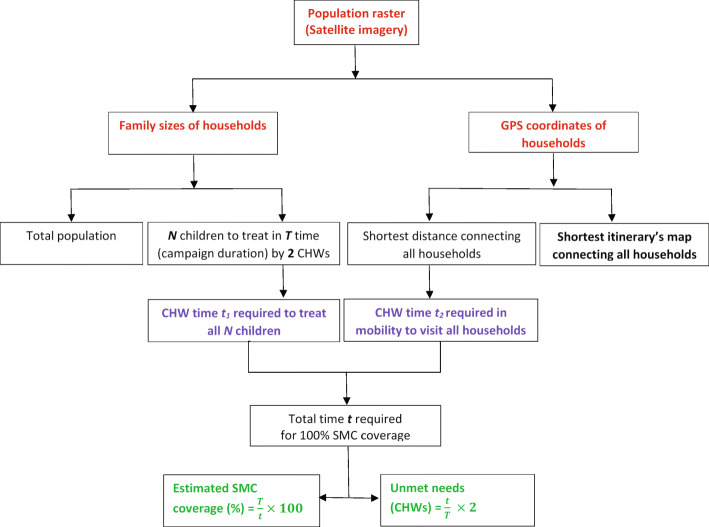
Microplanning model design

To improve SMC door-to-door delivery, we used a salesman algorithm-based accessibility model (Fig. 1) to determine the optimal itinerary for CHWs to efficiently visit all households in each village. The model computes the shortest door-to-door visit itinerary using global positioning system (GPS) information of households and outputs travel distance and travel time as well as treatment duration. Households’ GPS coordinates and family sizes were extracted from the population raster of each village. A 2015’s population raster of Burkina Faso as provided by the Center for International Earth Science Information Network (CIESIN) and the Connectivity Lab at Facebook encapsulates values on number of individuals inside raster’s pixels that can be processed to extract family sizes [19]. Fraction of under 5 children per household was subsequently derived from related-family size assuming that under 5 children make up approximately 18% of the total population [20].

#### Generating household visit itinerary using the salesman algorithm

We describe the traveling salesman problem (TSP) as a graph theory problem. Each household is thought of as a vertex and each vertex is connected by an edge, so our graphs is *G* = (*V*, *E*), where *V* is the set of vertices, and *E* is the set of edges. Each edge has an associated cost *c*_*ij*_, which is the distance between the two households. Our goal is to find the shortest path from any starting vertex that passes through all other vertices without repeating. Unlike the classic TSP, we do not return to the starting household. Instead, we use the Held-Karp algorithm, a dynamic programming solution [21]. The idea is to compute optimal sub-paths. We compute table entries C(S, i, j) for each subset S ⊂ V, and i, j ∈ S, defined to be the length of the shortest path from vertex *i* to vertex *j* visiting each vertex in *S* exactly once (and no node outside of *S*). The algorithm computes *C*(*S*, *i*, *j*) for increasing number of vertices in set *S*, up to *N*, the total number of vertices.

1. Let C({i, j}, i, j) = c_ij_ for all i ≠ j.
2. For *k* = 3 to *N*, do
  a For all sets S ⊂ V with *k* vertices, compute $C\left(S,i,j\right)=\underset{l\in S\backslash \left\{i,j\right\}}{\min}\left[C\left(S\backslash \left\{j\right\},i,l\right)+{c}_{lj}\right]$
3. Return the optimal cost $L=C\left(V,{n}_1,{n}_N\right)=\underset{i\ne j}{\min }C\left(V,i,j\right)$

We now can recover the path as follows: *n*_1_, *n*_*N*_ are our starting and ending vertices respectively. Vertex n_N − 1_ is the unique vertex satisfying

$$ \mathrm{C}\left(\mathrm{V},{\mathrm{n}}_1,{\mathrm{n}}_{\mathrm{N}}\right)=\mathrm{C}\left(\mathrm{V}\backslash \left\{{\mathrm{n}}_{\mathrm{N}}\right\},{\mathrm{n}}_1,{\mathrm{n}}_{\mathrm{N}-1}\right)+{\mathrm{c}}_{{\mathrm{n}}_{\mathrm{N}-1}{\mathrm{n}}_{\mathrm{N}}}. $$

If we have computed n_N − 1_, …, n_j + 1_, then vertex *n*_*j*_ is the unique vertex satisfying

$$ \mathrm{C}\left(\mathrm{V}\backslash \left\{{\mathrm{n}}_{\mathrm{N}},\dots, {\mathrm{n}}_{\mathrm{j}+2}\right\},{\mathrm{n}}_1,{\mathrm{n}}_{\mathrm{j}+1}\right)=\mathrm{C}\left(\mathrm{V}\backslash \left\{{\mathrm{n}}_{\mathrm{N}},\dots, {\mathrm{n}}_{\mathrm{j}+1}\right\},{\mathrm{n}}_1,{\mathrm{n}}_{\mathrm{j}}\right)+{\mathrm{c}}_{{\mathrm{n}}_{\mathrm{j}}{\mathrm{n}}_{\mathrm{j}+1.}} $$

We now have the whole path (*n*_1_, …, *n*_*N*_) along with optimal distance *L* = *C*(*V*, *n*_1_, *n*_*N*_).

With the path linking household via GPS coordinates, we could estimate walking distance using Euclidian distance formula.

#### Subdividing hard-to-reach areas using k-means clustering

For hard-to-reach villages with accessibility constraints (e.g. rivers), the model first clusters households using the constrained K-Means algorithm before determining optimal itinerary and unmet needs for each cluster [22].

We give the mathematical formulas for the constrained K-Means problem. Let the dataset be *D* = {*x*_1_, …, *x*_*m*_}, where *x*_*i*_ ∈ *R*^*n*^. Let 1 ≤ *k* ≤ *m* be the number of clusters. We want to find cluster centers *C*_1_, . . , *C*_*k*_ such that the distance between each point *x*_*i*_ and the nearest cluster center *C*_*h*_ is minimized under the condition that cluster number *h* must contain at least *τ*_*h*_ data points, where ${\sum}_{h=1}^k{\tau}_h\le m$. If *τ*_*h*_ > 0, this forces clusters to be non-empty, and we can also choose *τ*_*h*_ such that all clusters have relatively the same number of data points. We let T_i, h_ ∈ {0, 1} denote the “selection variables” that indicate whether *x*_*i*_ belongs to cluster number *h*. The constrained K-Means problem is as follows.

$$ \underset{C,T}{\min }{\sum}_{i=1}^m{\sum}_{h=1}^k{T}_{i,h}\left(\frac{1}{2}{\left\Vert {x}_i-{C}_h\right\Vert}^2\right) $$

We can solve this iteratively. At iteration *t*, let C_1, t_, …, C_k, t_ be the cluster centers. We compute the cluster centers C_1, t + 1_, …, C_k, t + 1_ at iteration t + 1 in 2 steps.

1. Cluster assignment: let ${\mathrm{T}}_{\mathrm{i},\mathrm{h}}^{\mathrm{t}}$ be a solution to the following linear program with C_h, t_ fixed
  $$ \underset{\mathrm{T}}{\min }{\sum}_{\mathrm{i}=1}^{\mathrm{m}}{\sum}_{\mathrm{h}=1}^{\mathrm{k}}{\mathrm{T}}_{\mathrm{i},\mathrm{h}}\left(\frac{1}{2}{\left\Vert {\mathrm{x}}_{\mathrm{i}}-{\mathrm{C}}_{\mathrm{h}}\right\Vert}^2\right) $$

$$ \mathrm{subject}\ \mathrm{to}\ {\sum}_{\mathrm{h}=1}^{\mathrm{k}}{\mathrm{T}}_{\mathrm{i},\mathrm{h}}=1,\mathrm{i}=1,\dots, \mathrm{m} $$

$$ \kern5.25em {\sum}_{\mathrm{i}=1}^{\mathrm{m}}{\mathrm{T}}_{\mathrm{i},\mathrm{h}}\ge {\uptau}_{\mathrm{h}},\mathrm{j}=1,\dots, \mathrm{k} $$

$$ \kern8.75em {\mathrm{T}}_{\mathrm{i},\mathrm{h}}\ge 0,\mathrm{i}=1,\dots, \mathrm{m},\mathrm{h}=1,\dots, \mathrm{k} $$

2. Update C_h, t + 1_as follows. If ${\sum}_{i=1}^m{T}_{i,h}^t=0$, then no update is made: *C*_*h*, *t* + 1_ = *C*_*h*, *t*_. If ${\sum}_{\mathrm{i}=1}^{\mathrm{m}}{\mathrm{T}}_{\mathrm{i},\mathrm{h}}^{\mathrm{t}}>0$, then
  $$ {\mathrm{C}}_{\mathrm{h},\mathrm{t}+1}=\frac{\sum_{\mathrm{i}=1}^{\mathrm{m}}{\mathrm{T}}_{\mathrm{i},\mathrm{h}}^{\mathrm{t}}{\mathrm{x}}_{\mathrm{i}}}{\sum_{\mathrm{i}=1}^{\mathrm{m}}{\mathrm{T}}_{\mathrm{i},\mathrm{h}}^{\mathrm{t}}} $$

We terminate when C_h, t + 1_ = C_h, t_ for all *h*. This algorithm is guaranteed to converge to a locally optimal solution. The constraints in the linear program in the cluster assignment step is equivalent to a Minimum Cost Flow (MCF) problem, a linear network optimization problem.

### SMC performance under current standard deployment

Current standard SMC deployment refers to as a door-to-door delivery performed by a CHW whose geographical orientation and time management is solely based on the CHW’s own perception. The number of CHWs for Rakolo during the 2016’s SMC campaign was limited to two who were trained by one supervisor (health facility nurse). MAPs were not carried by CHWs although rough sketched paper’s map was used by the supervisor to macro-plan SMC deployment across the health facility catchment area. SMC coverage was defined as follows: $SMC\  Coverage={\sum}_{\mathrm{i}=1}^{\mathrm{T}}\frac{n_i}{N}\times 100$ where T is campaign duration in days, *n* is number of treated and N the total number of children. Based on personal communications and on reports, we estimated at 12.5mn the average treatment duration per child ranging under 15mn to above 30mn in 63 to 22% of occasions respectively [23]. During the 2016’s SMC campaign, CHWs service packages were loaded with GPS devices and unknowingly provided GPS-tracking itineraries in Rakolo. Walking distances were estimated using visited household coordinates and converted to travel times assuming a 20mn walk per km in the wet season [24, 25].

### SMC performance under microplanning

To predict SMC coverage under microplanning, the model assumes an initial number of 2 CHWs, daily working time of 8 h, and 4 days of campaign duration. Walking distances in optimized visit itineraries were converted to travel times [24, 25]. Based on current CHWs experiences, we assumed random draws of treatment duration for 1, 2 or 3 children per household as follows t ∼ U (10, 15); t ∼ U (15, 20) or t ∼ U (20, 25) respectively. Assuming a household of 3 children we estimated on average 8.5mn (25mn/3) per child as best optimal treatment duration. Predicted SMC coverage was computed as follows: $SMC\  Coverage=\frac{\boldsymbol{T}}{\boldsymbol{t}}\times 100$ where T is campaign duration in days and t is total of treatment and travel times in days (Fig. 1).

We assessed CHWs’ performances (SMC coverages) and unmet needs under current SMC deployment mode and two microplanning scenarios (A and B). Microplanning A consists in optimizing visits itinerary and time invested in treatment while for microplanning B, visits itinerary, time invested in treatment and number of CHWs are optimized.

Comparison analyses of proportions of treated children and visited households between current SMC deployment and microplanning A or B were based on Chi^2^ test. Uncertainties around the optimal number for daily treated children and walking km were estimated as 95% Confidence Intervals using the t-distribution.

### Unmet needs for SMC performance maximization

To maximize SMC performance, unmet needs were estimated by converting maximum time invested to reach 100% of SMC coverage into number of CHWs needed:

Unmet needs (CHWs) = $\frac{t}{T}\times 2$ where T is campaign duration in days and t is total time invested for treatment and travel (Fig. 1). We chose to express unmet needs as supplementary CHWs instead of supplementary campaign days to reduce workforce burden (fatigue).

## Results

### Predicting population sizes using population raster

**Fig. 2:**
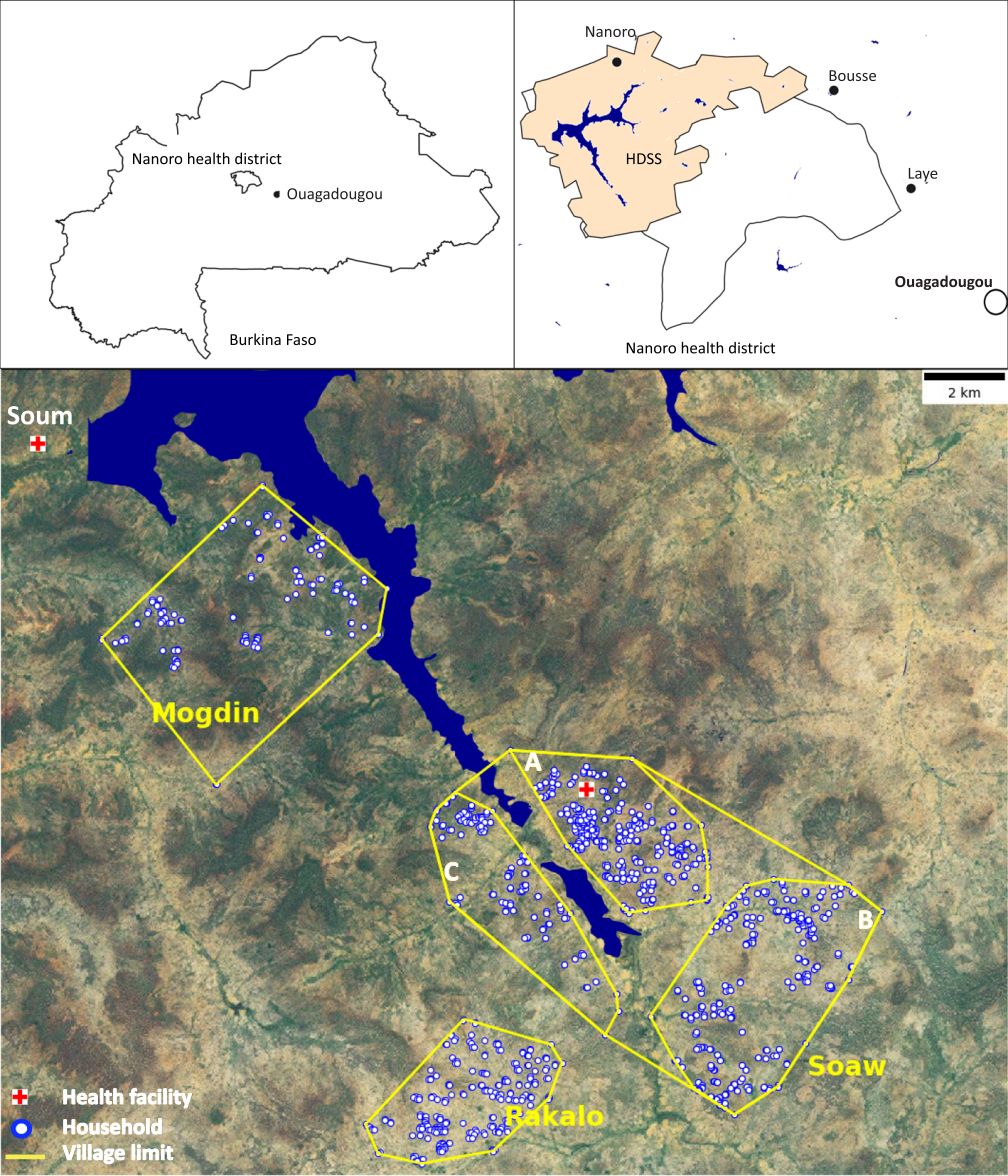
Study villages. Country and admin2 (Health District) MAPS were obtained form https://www.diva-gis.org/gdata. GPS coordinates of households were extracted from https://www.ciesin.columbia.edu/data/hrsl/

**Fig. 3:**
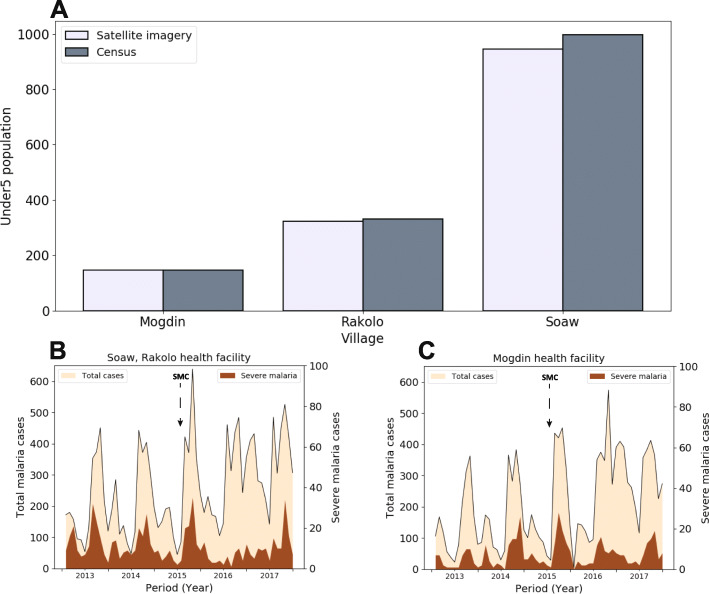
Population estimates and malaria burden in study villages

Three villages with varying characteristics were selected to assess CHWs’ performances and unmet needs under current SMC deployment mode and two microplanning scenarios (A and B). In Rakalo, households seem evenly spaced suggesting a uniform dispersion of the population (Fig. 2). Contrarily to Rakolo, households in Mogdin are unevenly spaced leading to a random dispersion of the population with longer distances to connect households (Fig. 2). In Soaw, households are distributed across water streams leading to clumped dispersion of the population suggesting that hard-to-reach scenarios should be accounted for while microplanning SMC delivery. We thus subdivided Soaw into three groups (A, B, C, Fig. 2) using k-means clustering. Predicted under 5 population sizes in Rakolo, Mogdin and Soaw using population raster were not significantly different (146, 324, 945) compared to census data (146, 331, 998 respectively; Fig. 3a).

### Malaria incidence not impacted by current SMC deployment

Routine incidence data of Soum’s primary health facility catchment area was assumed from at least both villages of Soum and Bogdin (Fig. 1). Data reported by Soaw primary health facility were assumed from at least both villages of Soaw and Rakolo. Note that routine data outside catchment area are likely reported by our health facilities and conversely but it is difficult to estimate a differential incidence data for each village. Temporal malaria incidence increased or stalled in all three villages as from 2013 to 2017 suggesting that SMC deployment has likely not been effective to date (Fig. 3b, c). Similar incidence trends were observed from other health facilities supporting that SMC is likely not impactful in the entire region (Supplementary Figure 1).

### Impact of microplanning on SMC coverage

**Fig. 4:**
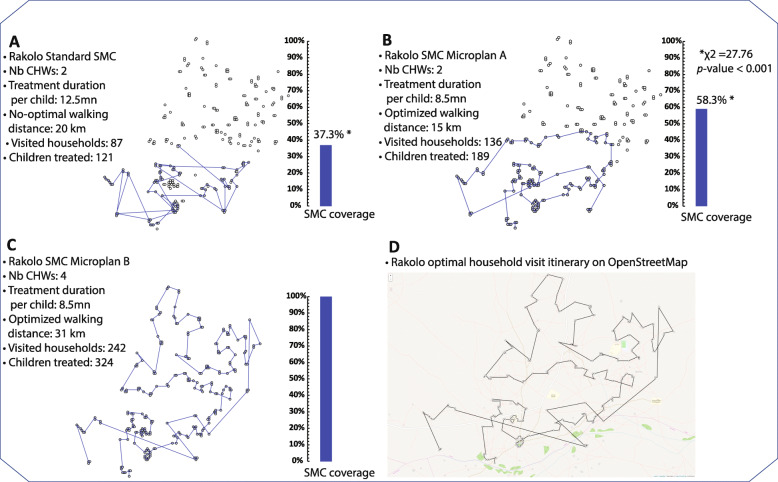
Performance of current SMC deployment mode (**a**) compared to microplanning (**b, c**) in Rakolo. Microplan A = Visits itinerary and time invested in treatment are optimized; Microplan B = Visits itinerary, time invested in treatment and number of CHWs are optimized

CHWs’ performance including number of visited households, number of treated children, walking distance and ultimately SMC coverage were estimated under current SMC deployment mode and compared to microplanning modes (Fig. 4a, b). Performances were predicted using population raster-based population estimates. Two types of microplanning (A, B), both assuming an optimal treatment duration of 8.5mn per child, were introduced to improve current SMC deployment. A microplanning A consisted of providing the CHW with a household visit itinerary plan which helps visualize the extent of the catchment area on a map with the shortest itinerary to visit all households. When microplanning A was predicted ineffective to cover 100% of households within the given campaign duration of 4 days, a microplanning B was introduced which consisted of optimizing (adjusting) the number of CHWs to timely cover all households and treatments.

Based on CHWs’ tracking data we predicted 37% of SMC coverage (Fig. 4a) in Rakolo which is in line with previous studies [11]. A total of 136 households were predicted to be visited by CHWs when an optimal visit itinerary plan was introduced (microplanning A, Fig. 4b) which significantly reduced CHWs’ walking distance by 25% (20 km to 15 km). This represents a significant increase by 36% (*χ2* = 19.15, *p* < 0.001) in the number of visited households as compared to the CHWs’ tracking data under current SMC deployment mode (87 households visited, Fig. 4a). The equivalent in SMC coverage was a significant increase by 21% (*χ2* = 27.76, *p* < 0.001, Fig. 4) as the number of children treated increased from 37.3% (121/324) under current SMC deployment mode up to 58.3% (189/324) following the introduction of optimal visit itinerary plan. Maximization of SMC coverage to 100% within 4 days window required 2 additional CHWs as unmet need in the roll out of microplanning B to extend the reach of SMC treatment (Fig. 4c). The practicality of using a model-optimized visit itinerary plan for SMC deployment in Rakolo is illustrated in Fig. 4d.

CHWs’ tracking data were not available for Mogdin and Soaw and therefore SMC coverage under current SMC deployment was not assessed. Microplanning A was systematically applied to Mogdin and Soaw and replaced by a microplanning B if 100% of SMC coverage was not reached under the former.

**Fig. 5:**
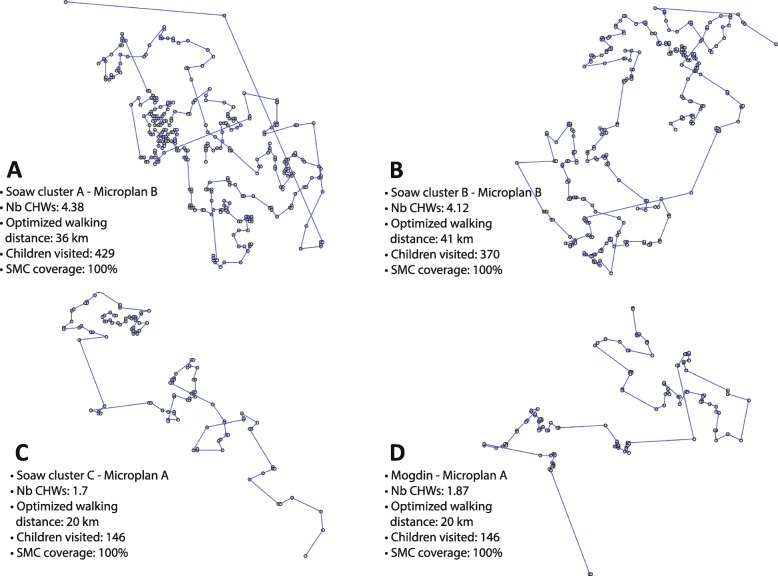
Performance of SMC microplanning in Soaw (**a, b, c**) and Mogdin (**d**). Microplan A = Visits itinerary and time invested in treatment are optimized; Microplan B = Visits itinerary, time invested in treatment and number of CHWs are optimized

In Soaw, many children in both clusters A (277 children) and B (235 children) were missed under microplanning A (Table 1). Unmet needs were estimated to be 2.38 and 2.12 additional CHWs required for cluster A and B respectively (Fig. 5a, b). In cluster C, an optimal visit itinerary alone (microplanning A) was enough to 2 CHWs to complete treatment of 146 children alongside 20 km of walking (Fig. 5c). Similarly, the use of an optimal visit itinerary alone in Mogdin was enough to cover all 146 eligible children in less than 4 days (Fig. 5d). The practicality of using a model-optimized visit itinerary plan for SMC deployment in Soaw is illustrated in Supplementary Figure 2.

**Table 1:** Performances of SMC under various deployment modes within 4 days of SMC campaign

Village	SMC Deployment mode	CHWs needed (Visited households)	Children treated (Treatment time)	Walking distance in km (Travel time)	SMC coverage (Treated/Total)	*P*-value
Rakolo	Current (Ref.)	2 (87)	121 (3.15 days)	20 (0.83 days)	37.3% (121/324)	
	Microplan A	2 (136)	189 (3.35 days)	15 (0.63 days)	58.3% (189/324)	< 0.001
	Microplan B	3.5 (242)	324 (5.74 days)	31 (1.29 days)	100% (324/324)	< 0.001
Mogdin	Microplan A	1.87 (126)	146 (2.58 days)	28 (1.16 days)	100% (146/146)	
Soaw A	Microplan A	2 (152)	195 (3.45)	13.2 (0.55 days)	45.6% (195/429)	
	Microplan B	4.38 (323)	429 (7.6 days)	36 (1.16 days)	100% (429/429)	
Soaw B	Microplan A	2 (135)	179 (3.16)	20.16 (0.84 days)	48.5% (179/370)	
	Microplan B	4.12 (278)	370 (6.55 days)	41 (1.70 days)	100% (370/370)	
Soaw C	Microplan A	1.7 (118)	146 (2.58 days)	20 (0.83 days)	100% (146/146)	
Average		2 (135)	178 (95%CI 108–248) (3.238 day)	19.03 (95%CI 12.9–25.1) (0.762 day)		

Current = Current SMC deployment mode on the ground, Microplan A = Visits itinerary and time invested in treatment are optimized; Microplan B = Visits itinerary, time invested in treatment and number of CHWs are optimized

On average we estimated that for a pair of CHWs, the daily optimal number of children for treatment and walking distance should not exceed 45 (95% CI 27–62) and 5 km (95% CI 3.2–6.2) respectively.

## Discussion

The present work identified opportunities to extend the reach of SMC treatment in rural African villages. Key inputs to SMC deployment including household visit itinerary, treatment duration and CHW ratio per capita were revisited under microplanning strategies. On average we estimated that for a pair of CHWs, the daily optimal average number of children for treatment and walking distance should not exceed 45 children and 5 km respectively over 4 days of campaign. We showed that optimal household visit itinerary reduces CHWs’ walking distance by 25%, increases the number of visited households by 36% and increases SMC coverage by 21% (37% under current standard mode up to 58% under microplanning). SMC coverage could be maximized to 100% only when the number of CHWs was adjusted to proportionate to the number of eligible children.

### Microplanning household visit itinerary

Our estimate of 37% of SMC coverage based on CHWs’ door-to-door tracking data is in line with previous findings [11] and possibly reflects current poor performance of SMC in the area. For millions of children in Sub-Saharan Africa, the door-to-door service delivery is a relief and often seen by stakeholders as an equity-focused strategy to leverage. In 2014, door-to-door delivery was deployed to maximize SMC coverage across the Sahel region, particularly to outreach those that are furthest from health facilities. But as with other interventions such as vaccination campaigns, the most vulnerable remain those that are remote and likely to be missed due to poor logistics or lack of microplanning. Our model estimates 21–63% increase in SMC coverage following the introduction of microplanning as 36% of households, previously missed by CHWs under current SMC deployment mode were recovered by microplanning. Using tracking systems and GIS-based microplanning, polio eradication teams were able to recover up to 38% of missed settlements from 8 Nigerian’s states during routine vaccine campaigns in 2013 [13] and an outreach of 31–43% (140 out of 322–441 settlements) during supplement immunization campaigns in Kano state [15]. Missing households or entire settlements during door-to-door service delivery is not uncommon in rural areas in Sub Saharan Africa where most households are not connected with roads. Without maps of catchment areas and having to crisscross winding trails and bushes, CHWs’ teams will not only walk longer distances but likely will miss households. A recent report highlights that the lack of planned itineraries and similarities between households, compounds contribute to confuse CHWs in their ability to identify the next household to be visited [14].

To be effective on the ground, SMC campaigns need to approach the excellence in delivery of clinical trials, but it must be practical and cost-effective. In clinical trials, households and participants are pre-identified and health workers trained to access them using rough sketch maps [26]. During campaigns, households are likely to be missed if biases in defining catchment areas are initially introduced in sketched paper maps or if such paper maps are not used that all [13, 15, 27].

The present work is on track to reproduce clinical trial delivery performance by focusing on tree basic needs for CHWs: go beyond rough sketching of settlements to use better microplanning of household visit itinerary, adjust CHW ratio based on population size and standardize treatment duration. Overall, our microplanning reduces treatment duration by 32% (12.5mn to 8.5mn per child) and can be standardized for better SMC performance. In Sub-Saharan Africa, census data are more often out of date or imprecise at sub-national levels while settlements identification [28–31] are not guaranteed to allow robust SMC deployment. Such biases are generally not accounted for during monitoring and evaluation efforts and often result in misleading SMC coverage estimates. The present work has the advantage to remotely assess family sizes [32] and adjust CHW ratio prior to SMC deployment. The work presents advantages of computing CHWs’ optimized travel itineraries and combining them with local accessibility features and geographies to generate printable accessibility maps or to incorporate such maps into mobile applications to be used by CHWs and supervisors.

### Limitations

Our work presents some limitations. We estimated population sizes of 2013 using a 2015 population raster which resulted in less uncertainties. For further estimates, updated population raster will be essential to capture population growth. The model optimizes SMC deployment on Day 1 only assuming that 2nd and 3rd SMC doses are well carried out by mothers on Day 2, 3. CHWs’ tracking data under current SMC deployment mode were only available for one village (Rakolo). More field data from villages with varying characteristics are needed to improve model robustness, including uncertainty assessment toward formal model validation. To predict SMC coverage, we did not account for fractions of kids vomiting, refusals or those not found at home (travelers or absence). Such data will be helpful in improving predictions of SMC coverage, and could be accounted for as they become available. Other challenges remain including reaching out to pastoralist and displaced populations due to conflicts. Finally, our salesman algorithm assumes catchment areas are free of physical barriers to generate free walking itineraries and therefore is currently not applicable to urban areas with excessive clustering. As next steps, we plan to include road - network processing to overcome such challenges in urban areas.

### Strengths, implications for other health programs, policy and practicality

The chronic underfunding of the health care system in low- and middle-income countries (LMCI) [33], has led to significant disparities in access to care across different settings. In Burkina Faso for instance the ratio of health infrastructure and of clinic visits per capita is estimated to be 1.03 (range 0.11–2.03) per 10,000 and 0.6 (range 0.08–1.01) per person per year respectively. Most deprived populations live in rural and remote settings [34]. Microplanning using satellite imagery and other geographic information systems [13, 15] are emerging strategies in door-to-door delivery and likely has recently contributed to wild polio eradication from Africa [35]. In the time of COVID-19, and to maximize the distribution of Insecticide Treated Net in COVID-19 affected countries, stakeholders for malaria prevention recently called out the need of microplanning strategies using topographic and route mapping [36] suggesting that our work may be helpful. As CHWs have emerged as critical human resources to health systems in LMCI, we believe that the present work might be an opportunity to assist and improve community health programs.

## Conclusion

To conclude, our work shows that microplanning contributes to extend SMC coverage by 21–63% in villages of Burkina Faso and may be reproducible elsewhere using free population raster. To the best of our knowledge, our work is first to assess opportunities of using microplanning strategies to improve SMC deployment on the ground. While this work focuses on SMC drug delivery, the microplanning strategy behind by addressing both households visit itinerary and CHW ratio per capita may have broader applicability for many other health service delivery programs such as vaccination, family planning or nutrition.

## Supplementary Information

**Additional file 1: Supplementary Figure 1.** Malaria incidence as reported by the Nanoro inpatient facility.

**Additional file 2: Supplementary Figure 2.** Optimized household visit itineraries over OpenStreetMap in Soaw.

**Additional file 3: Supplemental methods.** Extraction of households’ global positioning system (GPS) coordinates and family sizes from population raster.

## Data Availability

Data will be made available on reasonable request to the corresponding author

## Abbreviations

CIESIN: Center for International Earth Science Information Network
CHW: Community health worker
COVID-19: Coronavirus Disease 2019
DHIS2: District Health Information Software 2
GPS: Global Positioning System
HBHI: High burden to high impact
LMIC: Low- and Middle- Income Countries
SMC: Seasonal Malaria Chemoprevention
TSP: Traveling salesman problem
WHO: World Health Organization
