# Supplementary figures and images for: A microplanning model to improve door-to-door health service delivery: the case of Seasonal Malaria Chemoprevention in Sub-Saharan African villages
Source: BMC Health Serv Res. 2020 Dec 7;20:1128. doi: 10.1186/s12913-020-05972-2 (PMC7720067; doi:10.1186/s12913-020-05972-2)

## Inpatient health facility

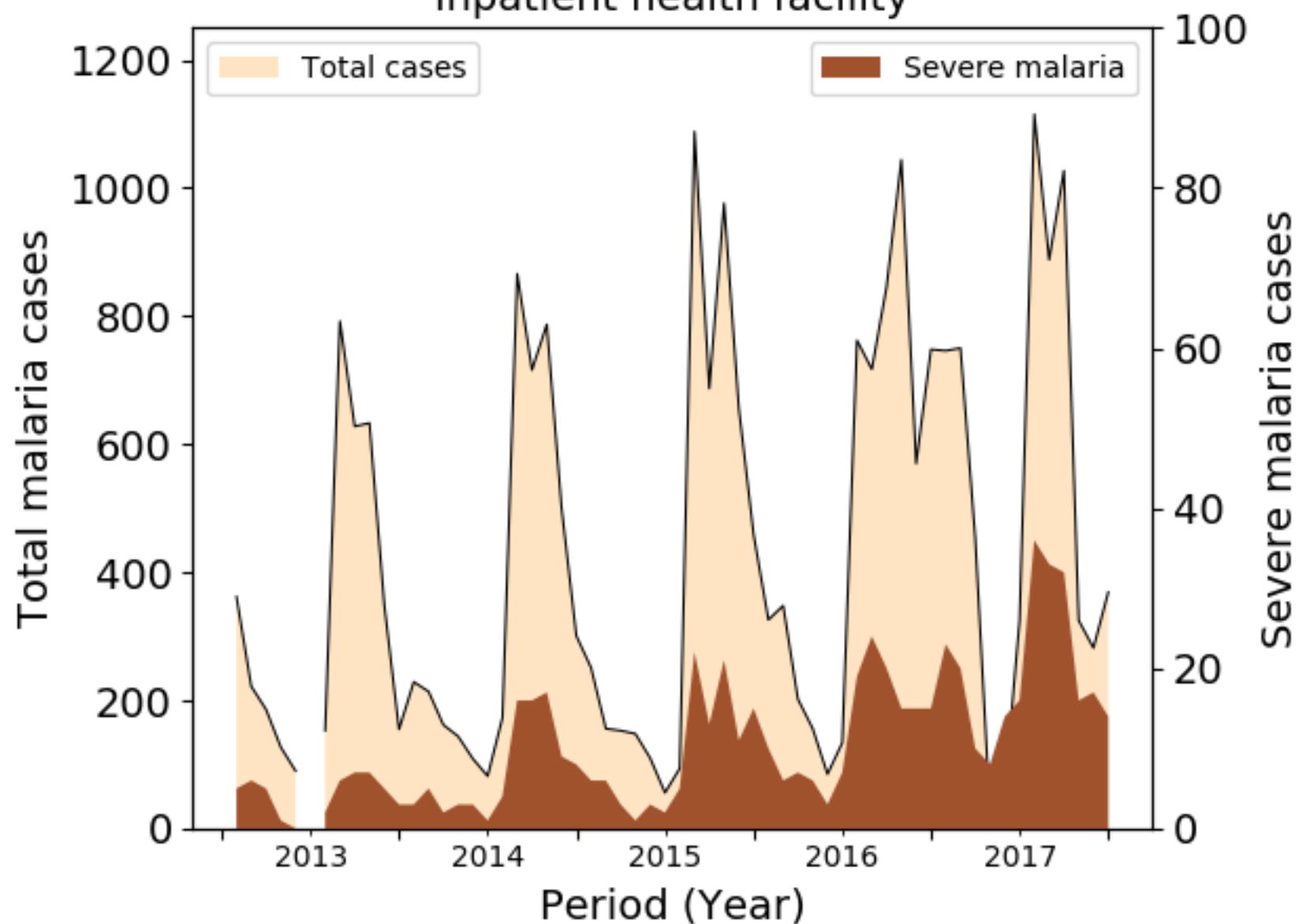

Supplement: Supplementary file 1 — Additional file 1: Supplementary Figure 1. Malaria incidence as reported by the Nanoro inpatient facility. [file 12913_2020_5972_MOESM1_ESM.pdf]

Soaw Cluster A

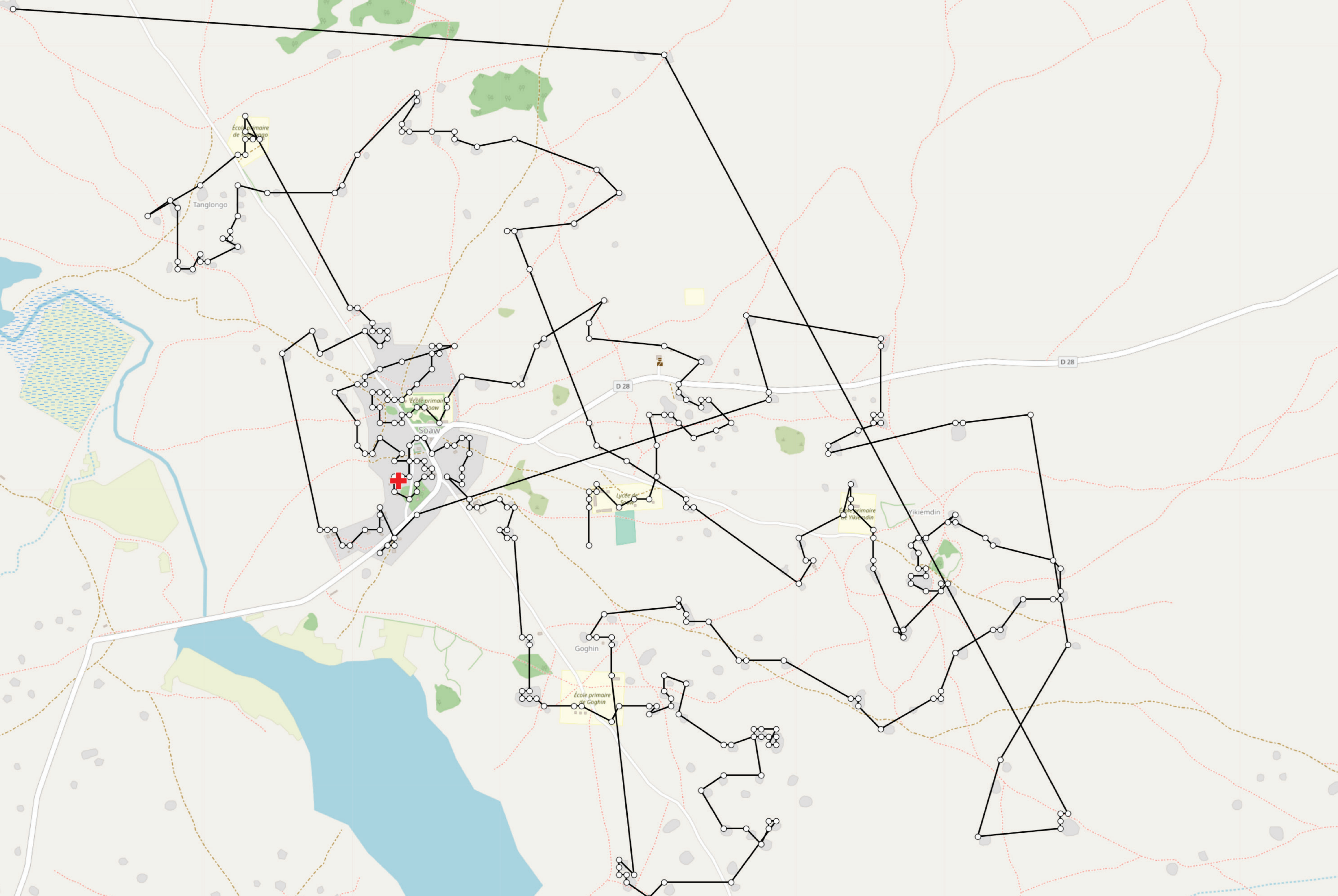

Soaw Cluster B

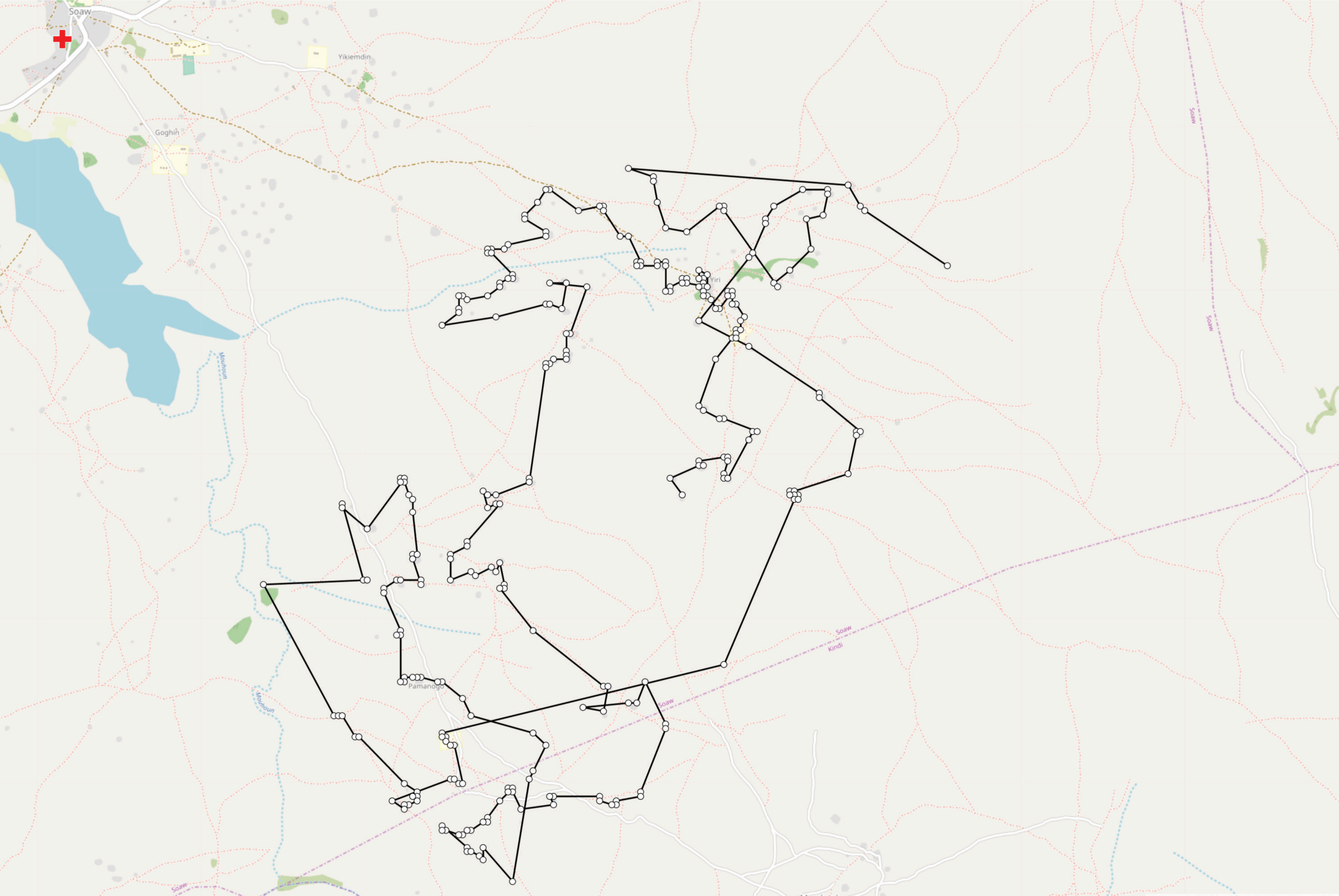

Soaw Cluster C

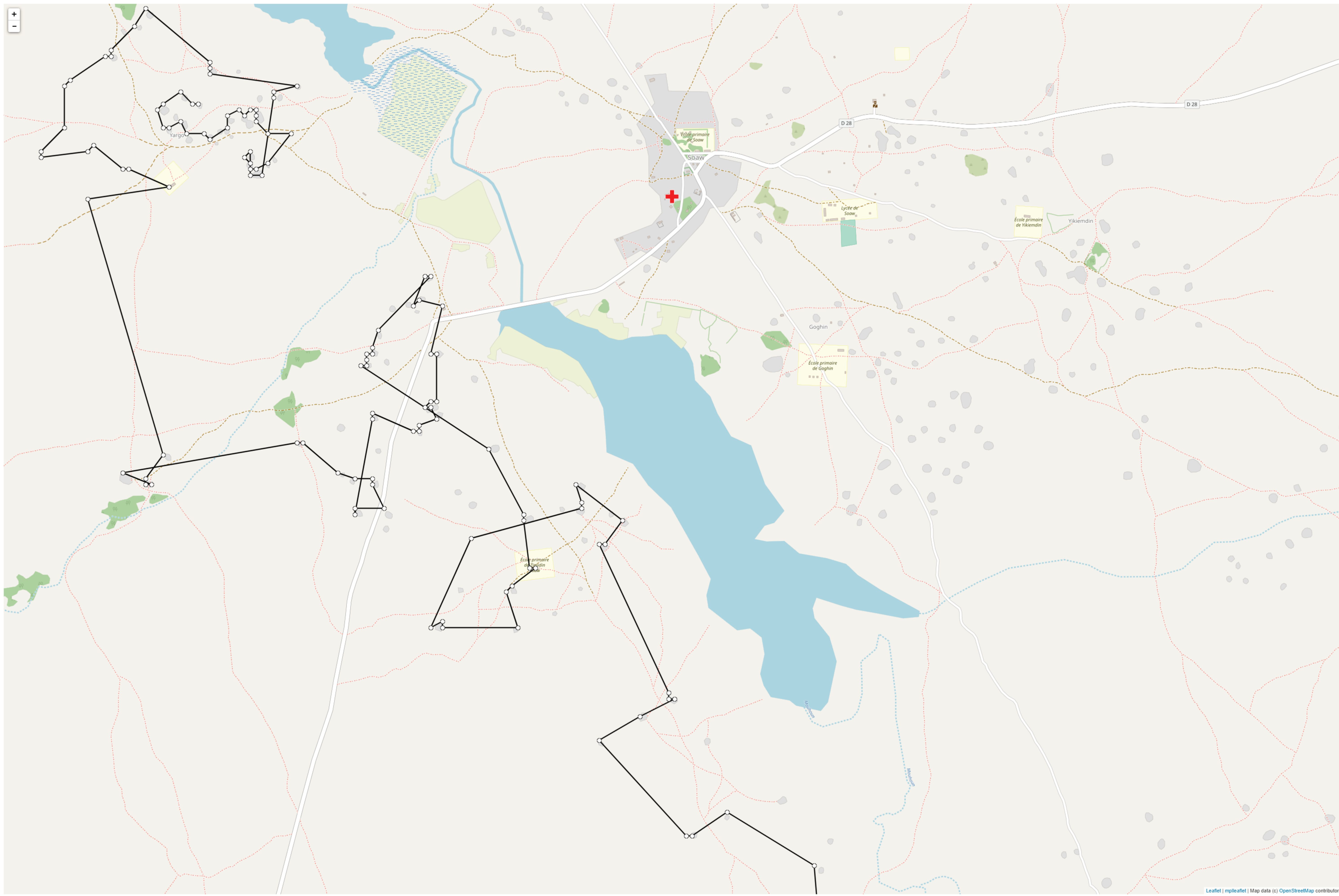

Supplement: Supplementary file 2 — Additional file 2: Supplementary Figure 2. Optimized household visit itineraries over OpenStreetMap in Soaw. [file 12913_2020_5972_MOESM2_ESM.pdf]
