# Supplementary material for: A microplanning model to improve door-to-door health service delivery: the case of Seasonal Malaria Chemoprevention in Sub-Saharan African villages
Source: BMC Health Serv Res. 2020 Dec 7;20:1128. doi: 10.1186/s12913-020-05972-2 (PMC7720067; doi:10.1186/s12913-020-05972-2)
Supplement: Supplementary file 3 — Additional file 3: Supplemental methods. Extraction of households’ global positioning system (GPS) coordinates and family sizes from population raster. [file 12913_2020_5972_MOESM3_ESM.docx]

A microplanning model to improve door-to-door health service delivery: The case of Seasonal Malaria Chemoprevention in Sub-Saharan African villages

André Lin Ouédraogo, Julie Zhang, Halidou Tinto, Innocent Valéa and Edward A. Wenger

***Supplemental methods:*** *Extraction of households’ global positioning system (GPS) coordinates and family sizes from population raster.*

***Aim:***

Extract population data from villages in Sub-Saharan Africa through the use of population raster

***Data extraction process:***

To extract GPS coordinates and family sizes of villages’ households, a population raster of Burkina Faso was downloaded from <https://www.ciesin.columbia.edu/data/hrsl>. The population raster is a part of a geospatial High-Resolution Settlement Layer (HRSL) developed jointly by the Center for International Earth Science Information Network (CIESIN) at Columbia University and the Connectivity Lab at Facebook [1]. It consists of a grid of pixels of particular sizes with discrete (x,y) locations and values and provides estimates of human population distribution at a resolution of 1 arc-second (approximately 30m) for the year 2015. More details can be found elsewhere [1]. Shapefiles are then used to clip population raster of villages of interest. Shapefiles are available or might be created otherwise. Methods using points coordinates or software such as ArcGIS (<https://www.arcgis.com/index.html>), QGIS (<https://www.qgis.org/en/site/>) can be used to generate shapefiles of interest.

Polygon-based shapefiles were used to clip population raster corresponding to each of the study villages (Rakolo, Soaw, Mogdin) and store as .TIFF fileS. A raster processing script was then used to extract population data including household size and GPS coordinate from file and stored for subsequent analyses. Our python script for raster data extraction can be read as follow:

'''Script to process population raster for coordinates and population data extraction'''

import os
import numpy as np
from osgeo import gdal
import rasterio
from rasterio.mask import mask
import geopandas as gpd
from shapely.geometry import mapping
from rasterio import Affine
import math

shapefile_of_interest = '.shp'
# # Dowload country's population raster from Connectivity Lab (Facebook)
country_population_raster= '.tif'
# Read shape file
shapefile = gpd.read_file(shapefile_of_interest)
# Geometry extraction as GeoJSON
geoms = shapefile.geometry.values
geometry = country_population_raster[0]
# Get GeJSON format
geoms = [mapping(geoms[0])]
# Extraction of raster values within polygon
with rasterio.open(country_population_raster) as src:
 out_image, out_transform = mask(src, geoms, crop=True)
no_data = src.nodata
# Extraction of values from masked array
data = out_image[0]
# Extraction of row, columns of valid values
row, col = np.where(data != no_data)
elev = np.extract(data != no_data, data)
# Reference pixel centre
T1 = out_transform * Affine.translation(0.5, 0.5)
rc2xy = lambda r, c: (c, r) * T1
population_data = gpd.GeoDataFrame({'col': col, 'row': row, 'household_size': elev})
# One could transform coordinates as follow
population_data['longitude'] = population_data.apply(lambda row: rc2xy(row.row, row.col)[0], axis=1)
population_data['latitude'] = population_data.apply(lambda row: rc2xy(row.row, row.col)[1], axis=1)
print (population_data)
# print or save_to_file (population_data)
population_data.to_csv('…file.csv')
print (file.head())
 col household_size row longitude latitude
0 66 6.0 21 -2.146529 12.738214
1 100 11.0 24 -2.137085 12.737381
2 110 6.0 26 -2.134307 12.736825
3 112 5.0 26 -2.133752 12.736825
4 111 7.0 27 -2.134029 12.736548

Reference:

1. Facebook Connectivity Lab and Center for International Earth Science Information Network (CIESIN) at Columbia University. High Resolution Settlement Layer (HRSL). <https://www.ciesin.columbia.edu/data/hrsl/>, 2016.
